# Whitening Effect of *Juglans regia* Dry Husk Extract on Primary and Permanent Teeth

**DOI:** 10.1155/2023/1037661

**Published:** 2023-07-21

**Authors:** Ola Hameed Turki, Zainab Juma Jafar

**Affiliations:** Department of Pedodontics and Preventive Dentistry, College of Dentistry, University of Baghdad, Baghdad, Iraq

## Abstract

Walnut is a common member of the family *Juglandaceae*. Recently, the evaluation of the phenolic content and antioxidant activity of the parts of walnut has received increased interest. Many reasons are responsible for teeth discolouration because teeth can absorb ingredients from tea, coffee, and food. Stains from these ingredients could stay in the porous enamel layer. Traditional whitening chemicals have some side effects, the most common of which is tooth sensitivity and mild or moderate gingival irritation. The aims of this comparative experimental study were to evaluate the whitening effect of *Juglans regia* dry husk extract and compare it with that of traditional prophylactic pumice. Forty human teeth were collected. Amongst these teeth, 20 were permanent, and the other 20 were primary. Each group was randomly divided into a study group (treated with dry husk extract) and a control group (treated with traditional pumice). Tooth colour was measured with Easy VitaShade Advanced 4.0, and the readings of lightness, chroma, and hue (*a*^*∗*^ represents the green to red axis and *b*^*∗*^ represents the blue to yellow axis) were documented for each tooth before and after polishing. A statistical analysis was performed using the Statistical Package for Social Science (version 22; Chicago, Illinois, USA). Data were analysed using Shapiro–Wilk, Wilcoxon sum rank, and Wilcoxon signed rank tests, and the level of significance was set to 0.05. A normality test was conducted using the Shapiro–Wilk test, and it showed that the colour variables were not normally distributed. With regard to tooth lightness, significant differences were observed in the primary and permanent teeth and *Juglans regia* exerted a much better whitening effect than pumice at *p* < 0.05. This study supports the use of *Juglans regia* dry husk extract in dentistry as a natural product with a whitening effect. It has utilisation potential in dentistry due to its beneficial properties and highly active components.

## 1. Introduction

People have many reasons for wanting to make their teeth white, and the most important one is cosmetic. Many commercial teeth whiteners are present containing synthetic chemical compounds, and long-term use could result in negative consequences because of gradual enamel erosion, which exposes the dentin [1]. For example, hydrogen peroxide causes gingival irritation and teeth sensitivity, and abuse of this material may lead to other critical adverse effects [2]. Numerous factors may cause teeth to become discoloured. Environmental variables, such as colours found in food, drinks, and medications (e.g., tetracycline), can cause extrinsic staining [3]. Smoking also contributes to teeth discolouration because tobacco poisons build up over time. As people age, their teeth become discoloured due to the buildup of various stains, and the enamel eventually dissolves to reveal the yellow dentin [4].

Plants have been utilised for medical purposes for several years, and self-medication using plants has become increasingly popular in recent years [5]. Some of these plants show potential, and natural materials are much better than chemical materials from simplicity and safety perspectives [6, 7]. *Juglans regia* is a traditional plant that has various names, including Persian walnut, common walnut, English walnut, Carpathian walnut, and Madeira nut, in many parts of the world [8]. The bark of this tree has been used as a teeth-cleaning twig due to its positive effects and the good feedback it has received in the field of oral health. It can clean the oral cavity and whiten teeth, which are commonly done in South Asia, North Africa, and the Middle East, and it can prevent the formation of calculus and bad breath. It also has various regional terms, including derum, dandasa, and sewak, in different populations [5].

## 2. Materials and Methods

Before conducting this *in vitro* study (comparative experimental study), the research protocol was approved by the Scientific Committee of the Pediatric and Preventative Department of the College of Dentistry, University of Baghdad, and by the Central Ethical Committee of the College of Dentistry, University of Baghdad (project number 474322).

### 2.1. Inclusion Criteria

The inclusion criteria were as follows: sound permanent premolars extracted for orthodontic purposes, sound primary incisors, and intact enamel of teeth with no caries or defects in the buccal surface.

### 2.2. Exclusion Criteria

Teeth with internal stains, decay, fluorosis, cracks, and restorations were excluded.

### 2.3. Sample Size and Sample Grouping

The sample of the present study consisted of 20 sound primary incisors (supplementary materials, Figure 1) and 20 sound human permanent premolar teeth (supplementary materials, Figure 2), which were selected based on specific selection criteria for orthodontic treatment from the Oral Surgery Department of the College of Dentistry, Baghdad University, and some private clinics in Baghdad City by using G power. The permanent teeth were divided into two subgroups randomly by using random team generator (splits a list into random groups) to guarantee randomisation. The primary teeth were divided in the same randomised manner.

### 2.4. Collection of *Juglans regia* Sample

Dry husk was collected from Al-Sulaymaniyah City in the north of Iraq (supplementary materials, Figure 3), and the sample was stored at room temperature in darkness for one month. Afterwards, extraction was performed to obtain active ingredients.

### 2.5. Extract Preparation

Dry husk (200 g) was ground into powder (supplementary materials, Figure 4), sieved until no large particles were present and mixed with 1 L of 70% ethanol (Applichem Gmbh Olloweg, Darmastdt, Germany) [9] in an ultrasonic device (RPM of 350; Kunshan Ultrasound, Jiangsu, China) at room temperature for 50 min. Materials needed for husk extraction are shown in supplementary materials, Figure 5. The resultant was inserted into a vacuum evaporator (Heidolph Instruments GmbH, Schwabach, Germany) to remove alcohol [10], and the final resultant was 250 ml of concentrated, heavy liquid (supplementary materials, Figure 6). The final resultant was lyophilised for 30 min until a paste-like consistency was obtained (supplementary materials, Figure 7) and placed in a closed glass container [11]. The resultant (20 g) was then used in the experiment in its full concentration in accordance with the results of a pilot study.

### 2.6. Measurement of Teeth Whiteness

Under standardised illumination conditions, colour measurement was performed with a calibrated spectrophotometer (VITA Easyshade®V, Zahnfabrik AG, Switzerland) and data from the *L*^*∗*^*A*^*∗*^*B* System (Commission Internationale de l'Eclairage). VitaShade was used to obtain accurate readings in the same conditions for all teeth before and after polishing. Daylight was excluded, and only one source of light was adopted for a specific box. The background was dark blue (supplementary materials, Figure 8), the teeth were fixated with artery forceps, and each tooth was in contact with the device at the measurement time. Three readings for *L*, *A*, *B*, *C*, and *H* were obtained in the same hour of the day (supplementary materials, Figure 9). All readings were repeated three times, averaged and recorded for a statistical analysis [12].

Hue is the quality that distinguishes one family of colour from another, and chroma is the saturation, intensity, or strength of a hue. Colour difference (∆*E*^*∗*^*ab*) is calculated as a function of a change in luminescence (∆*L*), with *L*^*∗*^ for lightness from black (0) to white (100), *a*^*∗*^ from green (−) to red (+), and *b*^*∗*^ from blue (−) to yellow (+). It is represented as ∆*E* [13], and the equation is

(1) $$\begin{array}{c} ∆E_{ab}^{*}=\sqrt{{\left(L_{2}^{*}-L_{1}^{*}\right)}^{2}+{\left(a_{2}^{*}-a_{1}^{*}\right)}^{2}+{\left(b_{2}^{*}-b_{1}^{*}\right)}^{2}}\text{.} \end{array}$$

### 2.7. Polishing Procedure

The teeth were divided into two groups. One group was polished with traditional nonfluoridated pumice (PD Dental, Vevey, Switzerland), and the other was polished with *J. regia* dry husk extract. The polishing procedure was performed with the help of an electric handpiece.

The electric handpiece was set to run at a speed of 8000 rpm, the torque of the device was set to 3.4 N-cm in accordance with the product information sheet [14] and the time was 10 s [15]. A single rubber cup was used for each tooth, and the teeth were held by artery forceps for fixation (supplementary materials, Figure 10). Instruments and materials needed for polishing procedure are shown in supplementary materials, Figure 11. The teeth were washed with water spray and dried with oil-free air for 10 s at each step. A 1 cm distance was utilised as a standard for holding the air water syringe away from the tooth surface. After polishing, the teeth were stored in distilled water.

## 3. Results and Discussion

A normality test was applied using the Shapiro–Wilk test, and it showed that the colour variables (*L*, *A*, *B*, *C*, and *H*) were not normally distributed amongst the groups and teeth at *p* < 0.05.

In the test on the changes in the colour variables (*L*, *A*, *B*, *C*, and *H*), significant differences in lightness (*L*) were observed in the primary and permanent teeth when *J. regia* dry husk extract was used. A significant difference in the change from green (−) to red (+), which represents *A*, was observed when prophylactic pumice was used in the primary teeth, as shown in Table 1.

The analysis of the colour change in the permanent teeth revealed a significant increase in *L* in the teeth treated with *J. regia* dry husk extract. *J. regia* dry husk extract showed no significant differences with prophylactic pumice in other colour variables, as shown in Table 2.

## 4. Discussion

Tooth colour is one of the most important characteristics that define our overall facial appearance, and it has a serious impact on psychological status, especially amongst young people. Brushing may not be enough, so dental or professional polishing may be necessary. Benefits can be obtained from natural, organic plants. Dentists must provide patients and communities with health education and keep them updated about the most effective and safe materials.

Research results show a promising future for nonchemicals because of the many advantages of natural materials, which may be superior to chemical materials from economic, safety, and simplicity perspectives.

Techniques for whitening teeth have gained increased popularity and concern from dental professionals due to the public's desire for white, bright teeth. Partial or whole-coverage restorations are challenging because of the fragility of the gingival margin in children and teenagers, thus degrading aesthetics.

The present study was designed to determine the efficacy of *J. regia* dry husk extract when used as a whitening material for teeth. When an electrical handpiece was utilised, the bias was reduced to the minimal level by controlling the device's torque, speed, and time; thus, these variables were the same for all samples [16]. The measurement of the amount of whitening difference was performed with VitaShade, which is considered an accurate device [17].

Colour has three parameters: hue, value, and chroma. Hue is the perception of the observer from the colour and depends on the different wavelengths of light beams that reach the eyes. Value is the achromatic dimension of colour and indicates its lightness/darkness. The higher the value is, the lighter the colour is, and vice versa. Chroma is the intensity of colour. The greater the chroma is, the richer the colour is [18].

The result of the comparison of pumice and *J. regia* dry husk extract showed that the latter is superior to the former from the whitening point of view (supplementary materials, Figures 13 and 15). This result is supported by those of other previous studies, which reported that the bark of this tree has a whitening ability [8]. *J. regia* contains juglone (5-hydroxy-1,4-napthoquinone), which is a chemical compound found predominantly in green husk and known as a tooth-whitening material [19]. The husk contains steroids and vitamins amongst other useful compounds, and the bark of this tree is tough and has been used for mechanical tooth cleaning due to its tough fibrous texture [20]. A change in the surface roughness of the enamel affects the lightness and green-red axis of tooth colour; when the surface is smooth, the colour is light [21].

Lightness (*L*) was increased in the primary and permanent teeth, but the increase in lightness was higher in the permanent teeth than in the primary teeth due to the fact that primary teeth are naturally whiter than permanent teeth [22]. The readings of *L* before the polishing procedure indicated that primary teeth had higher lightness than permanent teeth, so the total colour change was greater in permanent teeth. In addition, primary teeth have thin enamel, and because differences in enamel, dentin thickness, and mineral content already exist between primary and permanent teeth, the increase in the lightness of the primary teeth differed from that of the permanent teeth in this study [23]. Another reason may be related to the type of chosen teeth; the primary teeth in this work were incisors, whereas the permanent teeth were premolars. Premolars have a lower value of lightness compared with incisors [24].

Many explanatory variables, such as age, gender, coffee/tea consumption, and dental care, considerably affect yellowing (*b*^*∗*^) and brightness (*L*^*∗*^) [25]. A series of research has introduced *J. regia* dry husk extract as a polishing material and revealed additional favourable features, such as whitening and antibacterial effectiveness [26]. For this reason, a comparison was made with pumice.

Benefits can be obtained from natural plants, and the many advantages of these plants, such as safety, nontoxicity, and cost effectiveness, can be explored. Dentists always seek for the most updated product that will provide good results to improve the quality of treatment with no toxic or adverse effects. However, further *in vivo* studies are needed to determine if natural plants can be used effectively in the oral cavity and if the teeth and their supporting structures are negatively affected.

## 5. Conclusions

*J. regia* dry husk extract has a promising future in dentistry. The extract has a teeth whitening ability, as confirmed by this study. *J. regia* has abundant active components that support its utilisation. The data provided by this study serve as a foundation for biomaterial research and could facilitate the discovery of new clinically active natural product with teeth whitening effects, low cost, and no harmful effects.

### 5.1. Limitation of the Study

Further studies are needed to determine and examine the underlying mechanisms of the whitening effect of *J. regia* extract with this concentration and consistency directly *in vivo*.

## Figures and Tables

**Table 1:** Statistical test for the change in all colour variables (*L*, *A*, *B*, *C*, and *H*) in primary teeth.

Colour variables	Mean rank (pumice group)	*Z* (pumice group)	*p* value (pumice group)	Mean rank (*Juglans regia* group)	*Z* (*Juglans regia* group)	*p* value (*Juglans regia* group)
*L*	4.63	0.474	0.635	5.89	2.599	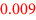
*A*	6.11	2.670	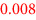	6.07	1.530	0.126
*B*	6.00	0.357	0.635	5.50	1.683	0.092
*C*	6.75	1.068	0.721	5.57	1.172	0.241
*H*	5.71	1.068	0.285	7.05	1.173	0.242

**Table 2:** Statistical test for the change in all colour variables (*L*, *A*, *B*, *C*, and *H*) in permanent teeth.

Colour variables	Mean rank (pumice group)	*Z* (pumice group)	*p* value (pumice group)	Mean rank (*Juglans regia* group)	*Z* (*Juglans regia* group)	*p* value (*Juglans regia* group)
*L*	5.67	0.663	0.507	6.00	2.090	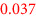
*A*	5.00	1.790	0.074	4.33	1.120	0.263
*B*	5.03	1.173	0.241	4.80	0.357	0.721
*C*	4.05	0.969	0.333	6.25	0.255	0.799
*H*	6.67	1.275	0.202	7.31	0.918	0.359

## Data Availability

The data used to support the findings of this study are available from the corresponding author upon request.

## Supplementary Materials

Supplementary Materials The supplementary file contains the pictures of different research steps; it was provided to enhance quality of the research.
