# Supplementary material for: Whitening Effect of Juglans regia Dry Husk Extract on Primary and Permanent Teeth
Source: Int J Biomater. 2023 Jul 21;2023:1037661. doi: 10.1155/2023/1037661 (PMC10382244; doi:10.1155/2023/1037661)
Supplement: Supplementary Materials — The supplementary file contains the pictures of different research steps; it was provided to enhance quality of the research. [file 1037661.f1.docx]

**Sample collection**


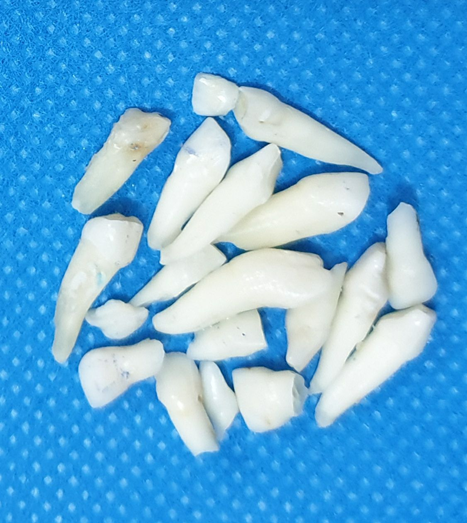


Figure 1: Sound primary incisor teeth.


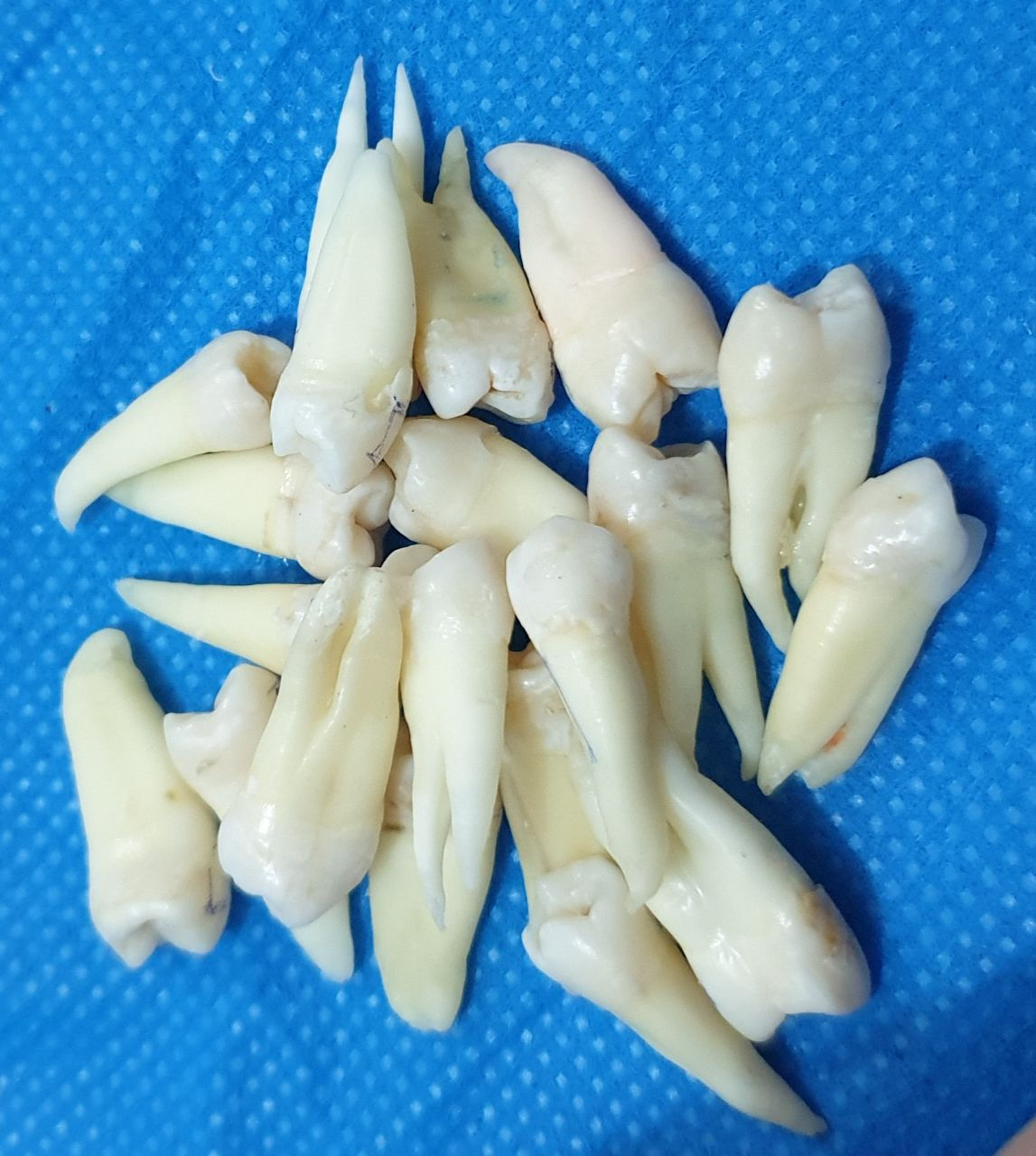


Figure 2: Sound permanent teeth.

**Juglans regia collection and extraction**


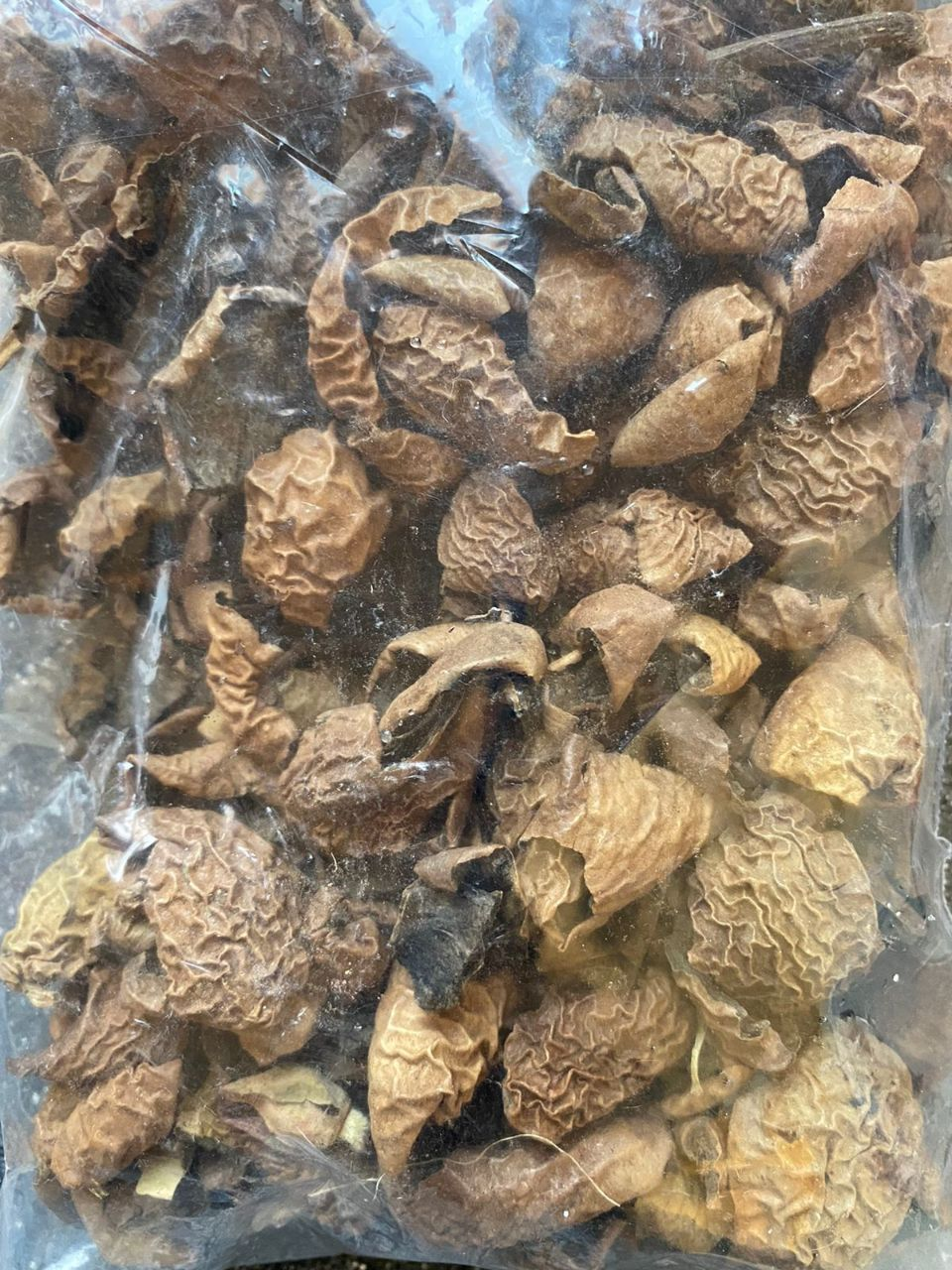


Figure 3: Collected J*uglans regia* dry husk.


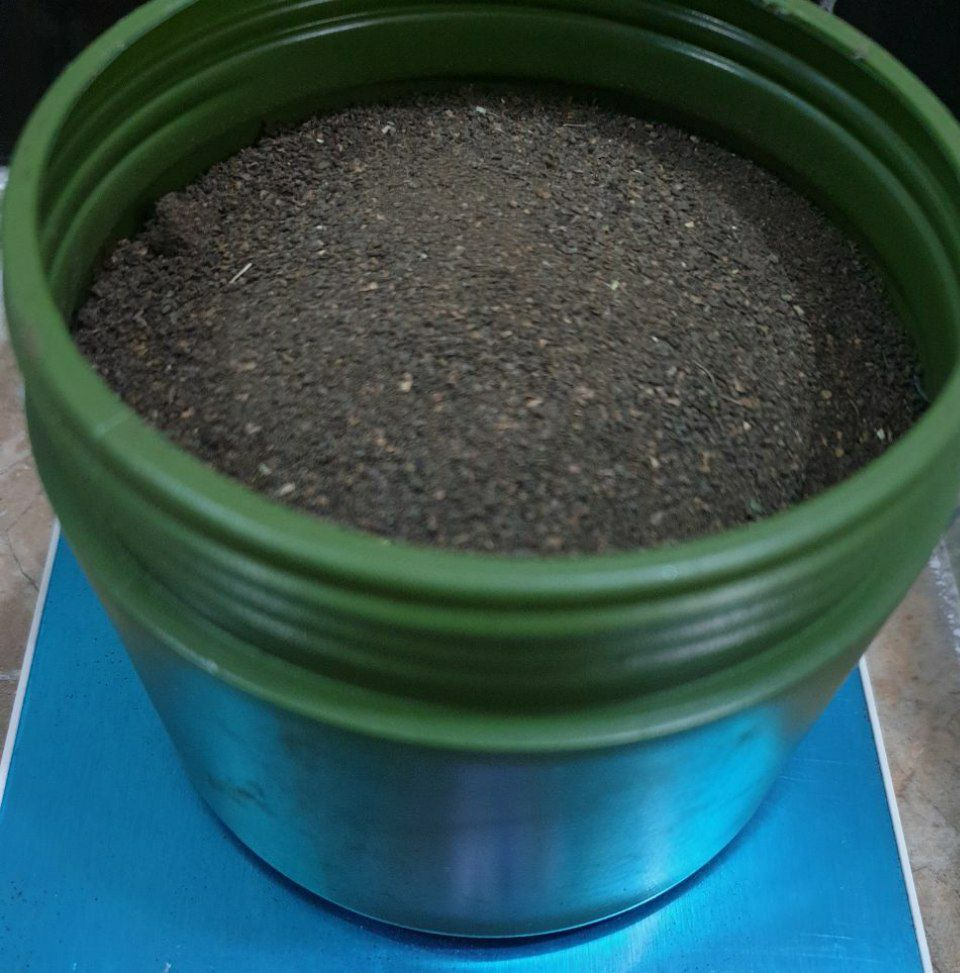


Figure 4: *Juglans regia* dry husk after being converted into powder.


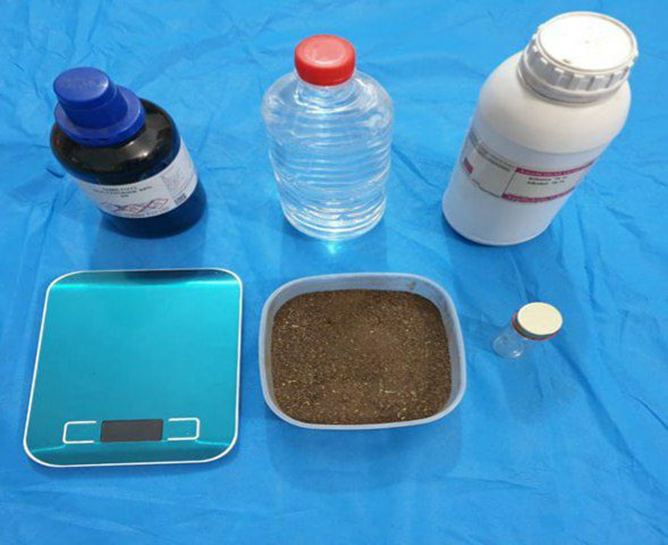


Figure 5: Materials needed for extraction.


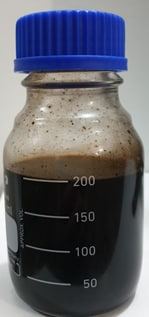


Figure 6: Heavy, concentrated liquid before lyophilisation.


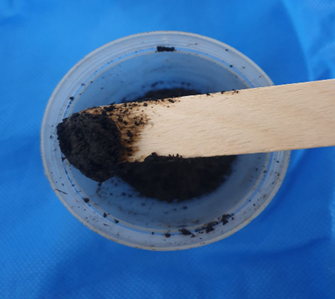


Figure 7: Final paste like consistency after lyophilisation.


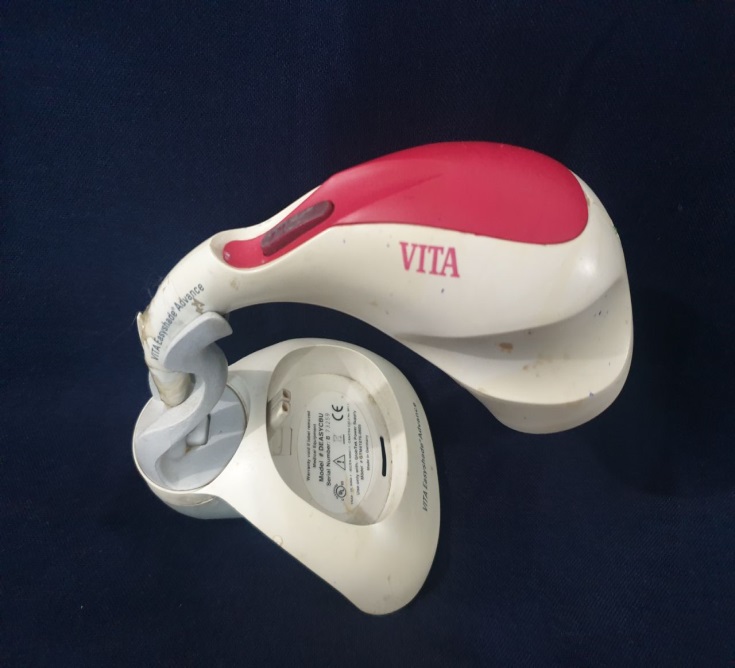


Figure 8: VITA Easyshade® calibration.


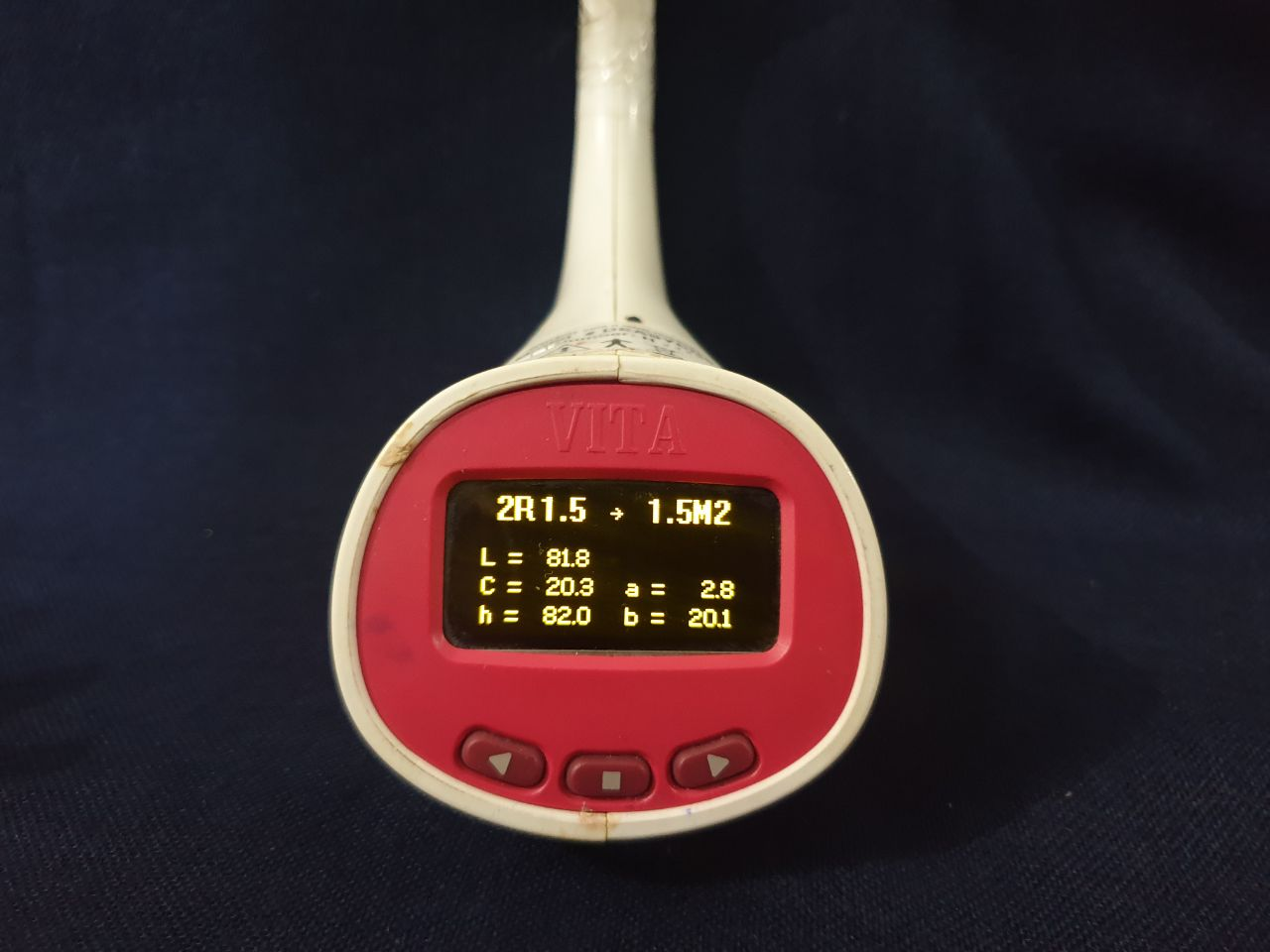


Figure 9: Recording the (L, A, B, C, H) by VITA Easyshade®.


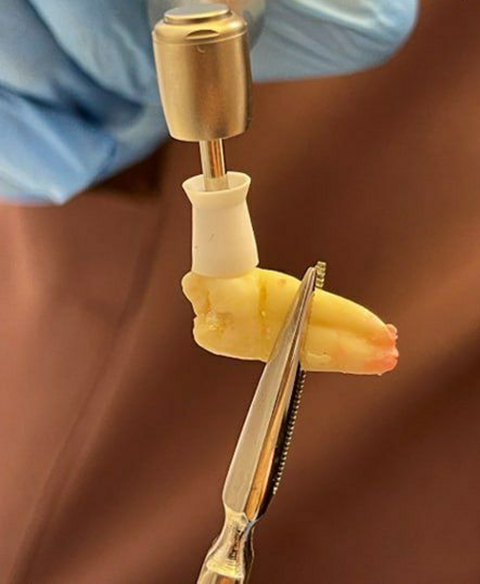


Figure 10: Polishing procedure, the tooth is fixed by artery forceps while polishing.


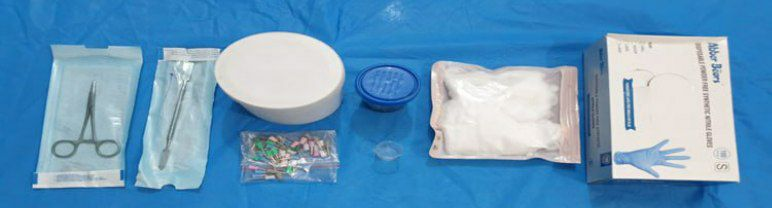


Figure 11: Instruments and materials needed for polishing procedure.

**Dgital, unmanipulated/unbiased images of the teeth both before and after treatment by both pumice and *Juglans regia.***


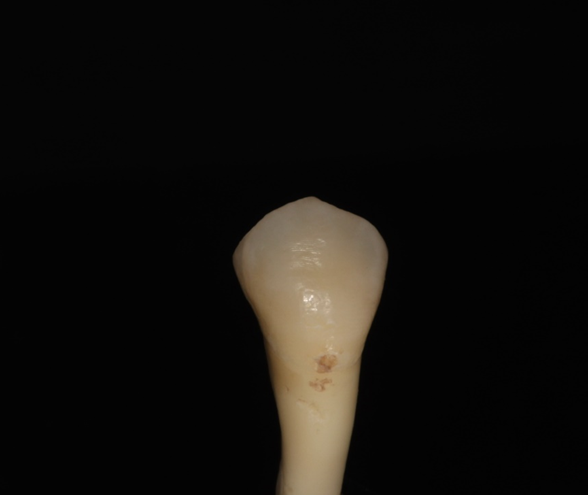


Figure 12: Upper second premolar before polishing with *Juglans regia* dry husk extract.


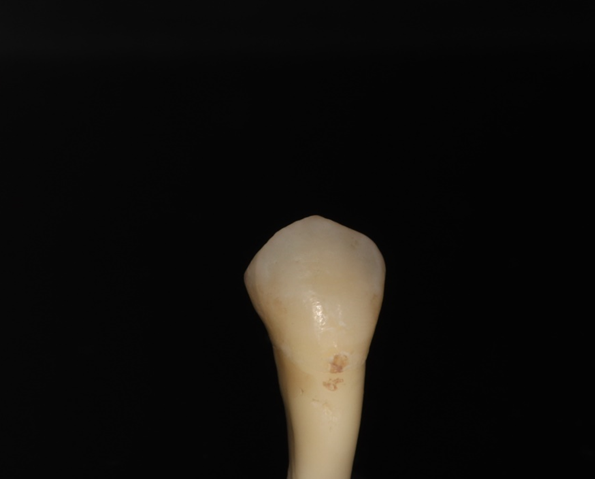


Figure 13: Upper second premolar after polishing with *Juglans regia* dry husk extract.


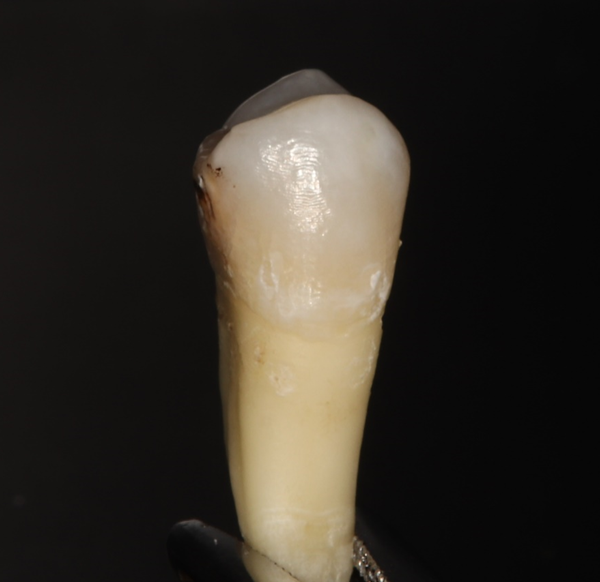


Figure 14: Upper second premolar before polishing with pumice.


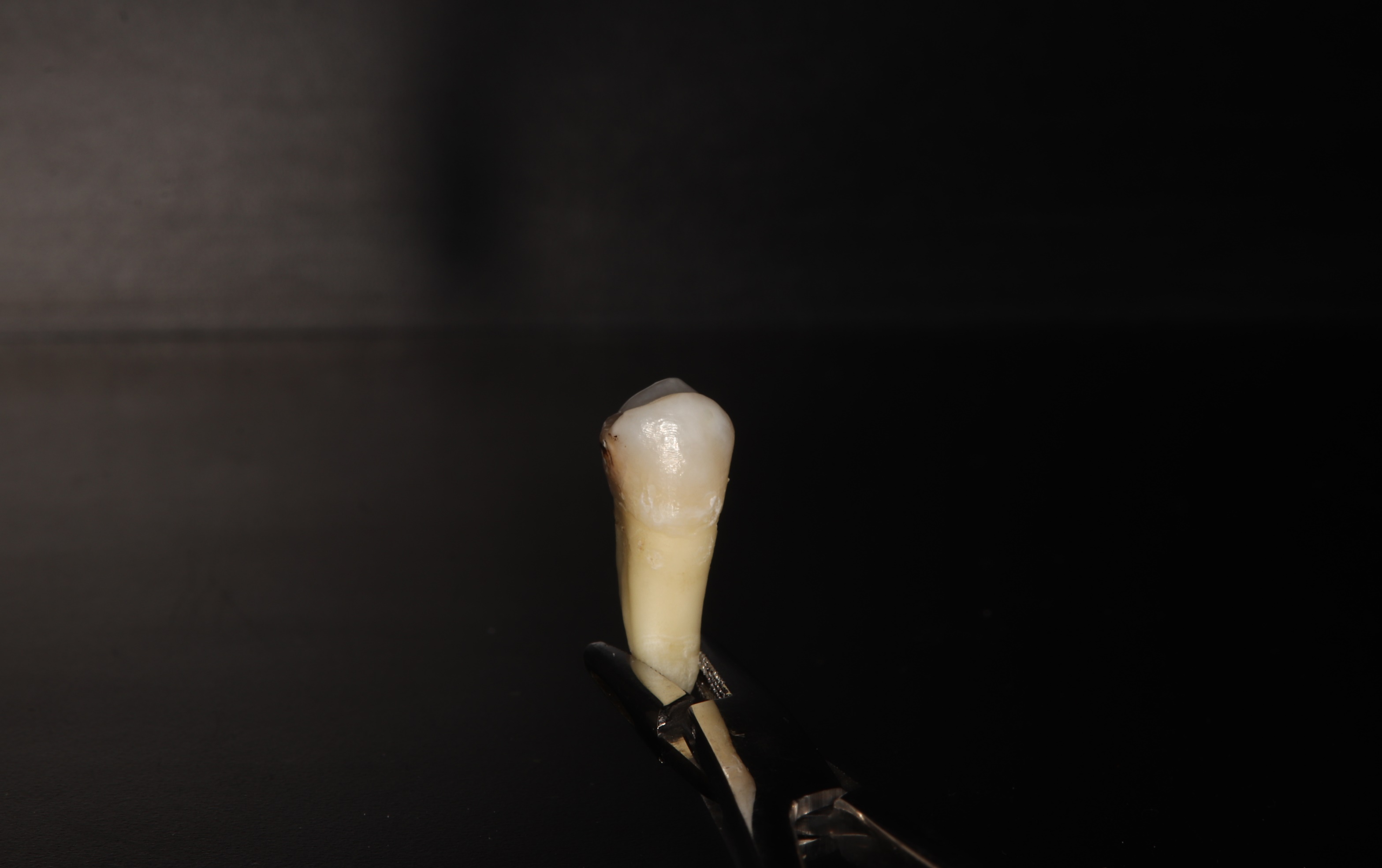


Figure 15: Upper second premolar after polishing with pumice.
